# Mitochondria in Renal Ischemia–Reperfusion Injury: From Mechanisms to Therapeutics

**DOI:** 10.3390/biomedicines14020310

**Published:** 2026-01-29

**Authors:** Yijun Pan, Jiefu Zhu

**Affiliations:** Department of Organ Transplantation, Renmin Hospital of Wuhan University, Wuhan University, Wuhan 430060, China; 2022305231076@whu.edu.cn

**Keywords:** renal ischemia–reperfusion injury, mitochondria, antioxidant defenses, mitochondrial quality control, mitochondrial DNA, mtDAMPs, mitochondria-targeted therapy, AKI

## Abstract

Renal ischemia–reperfusion injury (IRI) is a leading trigger of acute kidney injury (AKI), a syndrome with high incidence and mortality worldwide. The kidney is among the most energy-demanding organs; its mitochondrial content is second only to the heart, rendering renal function highly contingent on mitochondrial integrity. Accumulating evidence places mitochondria at the center of IRI pathogenesis. During ischemia, ATP depletion, ionic disequilibrium, and Ca^2+^ overload set the stage for injury; upon reperfusion, a burst of mitochondrial reactive oxygen species (mtROS), collapse of the mitochondrial membrane potential (ΔΨm), aberrant opening of the mitochondrial permeability transition pore (mPTP), mitochondrial DNA (mtDNA) damage, and release of mitochondrial damage-associated molecular patterns (mtDAMPs) further amplify inflammation and drive regulated cell-death programs. In recent years, the centrality of mitochondrial bioenergetics, quality control, and immune signaling in IRI-AKI has been increasingly recognized. Building on advances from the past five years, this review synthesizes mechanistic insights into mitochondrial dysfunction in renal IRI and surveys mitochondria-targeted therapeutic strategies—including antioxidant defenses, reinforcement of mitochondrial quality control (biogenesis, dynamics, mitophagy), and modulation of mtDAMP sensing—with the aim of informing future translational efforts in AKI.

## 1. Background

As a central organ for systemic metabolism and homeostasis, the kidney ranks second only to the heart in mitochondrial content and oxygen consumption [1]. Sustained ATP production is indispensable for tubular reabsorption, acid–base regulation, and electrolyte balance; consequently, renal function is tightly linked to both the number and quality of mitochondria. Substantial evidence implicates mitochondria across diverse renal pathologies, including ischemia–reperfusion injury [2], drug- or infection-induced AKI [3,4], chronic kidney diseases such as diabetic kidney disease [5], hypertensive nephropathy [6], and obstructive nephropathy [7], as well as renal neoplasms [8] and selected genetic kidney disorders [9].

Mechanistically, renal IRI features ischemic ATP depletion, ionic disequilibrium, and Ca^2+^ overload, followed—on reperfusion—by mtROS surges, ΔΨm collapse, pathological mPTP opening, mtDNA injury, and mtDAMP release. These events converge on cell-death pathways and sterile inflammation, culminating in tissue dysfunction. Against this backdrop, mitochondria have emerged as actionable therapeutic targets in IRI-AKI, motivating the systematic appraisal presented in this review.

## 2. Mitochondria in Renal Ischemia–Reperfusion Injury

Acute kidney injury (AKI) is a multifactorial syndrome affecting 10–15% of hospitalized patients and over 50% of those in intensive care units (ICUs) worldwide [10]. Renal ischemia–reperfusion injury (IRI) is a prototypical cause of AKI, precipitated by trauma, shock, kidney transplantation, and cardiovascular surgery. Transient interruption of renal blood flow restricts oxygen delivery; paradoxically, re-establishing perfusion amplifies tissue damage through a cascade of injurious mediators rather than restoring function [11]. Mitochondria have emerged as central regulators of these processes. Beyond serving as the primary site of oxidative phosphorylation (OXPHOS) for adenosine triphosphate (ATP) synthesis, mitochondria govern calcium homeostasis, heme biosynthesis, and signaling programs that control cell proliferation and apoptosis. As double-membrane organelles, their function depends on the structural and biochemical integrity of the outer mitochondrial membrane (OMM) and inner mitochondrial membrane (IMM). The OMM, enriched in porins and associated enzymes, mediates exchange with the cytosol, whereas the protein-dense IMM houses transporters and the electron transport chain (ETC), supporting aerobic respiration. Electrons supplied by NADH and FADH_2_ from the tricarboxylic acid (TCA) cycle enter the ETC through complex I or II, are transferred to ubiquinone (coenzyme Q), then to complex III, cytochrome c, and complex IV, where molecular oxygen is reduced. Proton pumping by complexes I, III, and IV generates the mitochondrial membrane potential (ΔΨm), which drives H^+^ flux through complex V (ATP synthase) to phosphorylate ADP and produce ATP [12,13].

The mitochondrial pathobiology of IRI unfolds across ischemic and reperfusion phases. During ischemia, oxygen deprivation suppresses OXPHOS and depletes ATP; intracellular H^+^ accumulation slows or reverses the Na^+^/Ca^2+^ exchanger, promoting cytosolic Ca^2+^ overload [14]. Upon reperfusion, an early burst of mitochondrial reactive oxygen species (mtROS) occurs, driven by damage to ETC components and rapid oxidation of succinate accumulated during ischemia—classically via reverse electron transport at complex I [11,15]. Ca^2+^ overload and mtROS synergize to intensify oxidative stress, degrade membrane proteins and phospholipids, and injure mitochondrial DNA (mtDNA) [16,17]. These perturbations favor sustained opening of the mitochondrial permeability transition pore (mPTP) in the IMM [18,19], causing ΔΨm collapse, organelle swelling, and membrane rupture. Through the mPTP, multiple pro-apoptotic factors and damage-associated molecular patterns (DAMPs) are released; fragmented oxidized mtDNA can transit the mPTP and voltage-dependent anion channel (VDAC) to the cytosol, activating the NLRP3 inflammasome and the cGAS–STING pathway and thereby amplifying inflammation [20]. In parallel, mitochondrial outer membrane permeabilization (MOMP) contributes decisively to apoptosis in IRI-AKI: increased OMM permeability enables release of cytochrome c and other pro-apoptotic effectors to engage the caspase cascade [21,22]. In mouse models of ischemic AKI, genetic deletion of Bak and Bax prevents MOMP and markedly attenuates apoptotic cell death [22].

Programmed cell death (PCD) pathways—including apoptosis and regulated necrosis (necroptosis, pyroptosis, ferroptosis)—constitute the predominant modes of tubular cell demise in AKI [23]. Upstream mitochondrial events—Ca^2+^ overload, oxidative stress, and aberrant mPTP opening—converge to dictate these outcomes. Accordingly, mitochondrial-targeted interventions that limit ROS generation, stabilize Ca^2+^ handling, restrain mPTP opening, or otherwise preserve organellar integrity consistently reduce tubular injury and cell death in experimental models [24,25,26]. Collectively, restoring mitochondrial function and maintaining mitochondrial architecture represent promising therapeutic strategies for IRI-AKI.

**Figure 1:**
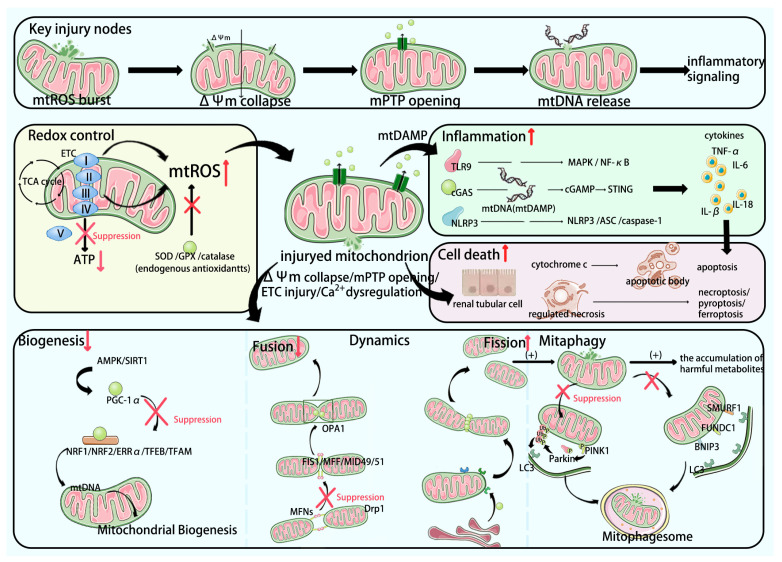
Integrated overview of interconnected mitochondrial dysfunction pathways in renal IRI-AKI. Ischemia/reperfusion triggers Ca^2+^ overload and mtROS surges, promoting ΔΨm collapse, mPTP opening, MQC imbalance (biogenesis–dynamics–mitophagy), and release of mtDAMPs (e.g., mtDNA/mtRNA and cardiolipin). These signals activate innate sensors (e.g., TLR9, cGAS–STING, and NLRP3) and amplify inflammation and regulated cell death; multiple therapeutic entry points include antioxidants, biogenesis activators, fission/fusion modulators, mitophagy tuning, and mtDAMP-pathway inhibition. (Red upward arrows indicate activation of the process, while downward arrows indicate inhibition.).

Figure 1 presents an integrated overview of the interconnected processes of mitochondrial injury, including mitochondrial quality control (MQC) failure, mtDAMP release, and immune activation, along with the key pathways involved.

## 3. Renal Mitochondrial Morphology

Advances in imaging and spatial-omics have clarified that renal mitochondria are far from homogeneous: their morphology, density, and ultrastructure vary markedly across nephron segments, tightly matching segment-specific transport workloads. Because proximal tubular epithelial cells (RPTCs) reabsorb ~80% of glomerular filtrate to maintain volume and solute homeostasis, they are widely considered especially susceptible to mitochondrial dysfunction [13]. Using an integrated pipeline combining spatial and single-cell transcriptomics, immunofluorescence, and isotope-tracing microscopy, Arnoux et al. recently identified the outer-medullary thick ascending limb (TAL) as a principal mitochondrial “injury hotspot” in AKI [27]. Cells within these energetically demanding regions contain abundant, elongated mitochondria whose inner membranes form densely packed, lamellar cristae. Such cristae expansion greatly increases IMM surface area, enabling high packing of ETC complexes and ATP synthase to sustain OXPHOS; however, these same features render the TAL exquisitely vulnerable to hypoxia-reoxygenation, with the most pronounced structural and functional damage during IRI [28]. In vivo real-time imaging in rat IRI-AKI has visualized acute mitochondrial swelling and fragmentation within tubular cells [29]. Complementing these observations, Saeki et al. employed an 18F-BCPP-BF PET probe targeting complex I and showed that, as early as 3 h after reperfusion in a rat renal IRI model, mitochondrial functional signals were already markedly diminished in key regions such as the outer medulla—even though total mitochondrial protein abundance had not yet declined. Electron microscopy from the same regions confirmed conspicuous mitochondrial swelling, underscoring the utility of PET to capture pathophysiologic events that precede conventional readouts and to provide an early window for risk stratification and intervention [30].

Crucially, mitochondrial impairment is not an isolated lesion. As the cellular hub for metabolism and signaling, mitochondria engage in intimate cross-talk with other organelles and structural systems [31,32]. At mitochondria–ER contact sites, lipid exchange and Ca^2+^ transfer are physiologic; in IRI-induced Ca^2+^ overload, however, these interfaces can precipitate mPTP opening and cell death [33]. Coordinated action with lysosomes governs mitophagy; its dysregulation in IRI promotes accumulation of damaged mitochondria and toxic metabolites [34]. Emerging evidence indicates that lysosomal dysfunction—impaired acidification, reduced hydrolase activity, or defective autophagosome–lysosome fusion—can become a rate-limiting step that stalls mitophagic flux in renal IRI. In such settings, autophagosomes may form but fail to complete degradation, leading to persistence of fragmented, ROS-producing mitochondria and amplification of inflammatory signaling. Activation of the TFEB-CLEAR lysosomal biogenesis program (e.g., urolithin A-mediated TFEB activation) has been reported to restore autophagic flux and attenuate renal ischemia–reperfusion injury in mice [35]. These findings highlight the therapeutic relevance of the mitochondria–lysosome–ER axis, suggesting that restoring lysosomal competence and organelle contact-site homeostasis may synergize with mitochondria-targeted antioxidants and MQC modulators. In parallel, mitochondria relay retrograde signals to the nucleus and exploit the cytoskeletal network for precise subcellular positioning to match local ATP demand [36,37]. Accordingly, mitochondrial injury in renal IRI propagates through these interconnected networks to drive a systems-level collapse of cellular homeostasis.

## 4. Mitochondrial Antioxidant Defense

Under ischemia–reperfusion injury (IRI), mitochondria are highly susceptible to multifaceted insults that precipitate cell death. A surge of mitochondrial reactive oxygen species (mtROS) is widely regarded as a pivotal event during this period. mtROS has been shown to compromise the ability of mitochondrial transcription factor A (TFAM) to safeguard mitochondrial DNA (mtDNA), thereby aggravating mitochondrial dysfunction and inflammation [17]. In renal IRI, a core pathogenic axis is the imbalance between ROS production and clearance. Members of the NADPH oxidase (NOX) family are activated and, by consuming NADPH, generate large amounts of ROS [38]. Multiple studies demonstrate that selective NOX inhibition effectively suppresses ROS overproduction and significantly attenuates subsequent renal injury [39,40]. More recently, remote ischemic preconditioning (rIPC) was reported to dampen NOX4–ROS signaling, thereby mitigating mitochondrial dysfunction and ferroptosis in tubular epithelial cells during AKI [41]. In parallel, endogenous antioxidant enzymes—superoxide dismutase (SOD), glutathione peroxidase (GPX), and catalase—normally restrain ROS and limit oxidative stress; however, their expression is consistently downregulated in IRI, leading to further ROS accumulation [42].

Excess mtROS damages respiratory-chain complexes and peroxidizes membrane phospholipids, establishing a self-amplifying vicious cycle. Accordingly, exogenous antioxidants and mitochondria-targeted redox modulators can quench ROS, curb lipid peroxidation, and preserve mitochondrial function. Several mitochondrial-directed antioxidants have shown promise, including MitoQ, SkQ, ubiquinone (CoQ10), and curcumin [43,44,45]. The mitochondria-targeted antioxidant peptide SS-31 markedly lowers mtROS in ischemic AKI models and, by binding cardiolipin, stabilizes inner mitochondrial membrane architecture [46,47]. Beyond small molecules, nucleic-acid–based strategies are emerging: lipid-nanoparticle (LNP) delivery of chemically modified SOD2 mRNA conferred renoprotection in murine renal IRI [48]. Similarly, Lei et al. engineered thylakoid–liposome biomimetic vesicles loaded with L-ascorbic acid; following intravenous administration with local renal ultrasound, this platform restored tubular bioenergetics and antioxidant capacity and improved renal function in experimental AKI, offering a new direction for IRI therapy [49]. Representative antioxidant/redox interventions are summarized in Table 1.

## 5. Mitochondrial Quality Control

If antioxidant defenses constitute the mitochondrion’s front-line response to stress, mitochondrial quality control (MQC) is its maintenance system. To stabilize mitochondrial number and function, the MQC network—coordinately regulated by nuclear and mitochondrial gene programs—orchestrates a highly dynamic continuum that governs mitochondrial morphology, clears damaged components, and repairs dysfunctional units, thereby sustaining the health of the mitochondrial network [50]. Core MQC modules include mitochondrial biogenesis, mitochondrial dynamics (fusion and fission), and mitophagy, which act in concert to maintain mitochondrial homeostasis under physiologic and pathologic conditions.

In ischemia–reperfusion injury (IRI), disruption of any MQC node destabilizes mitochondrial quantity and performance, precipitating bioenergetic failure, excessive ROS, and downstream cell death programs. Pharmacologic strategies that target MQC pathways have demonstrated efficacy across multiple organs [51,52], and in renal IRI they likewise attenuate tubular injury and improve renal recovery, underscoring MQC as a promising therapeutic axis.

### 5.1. Mitochondrial Biogenesis

**Figure 2:**
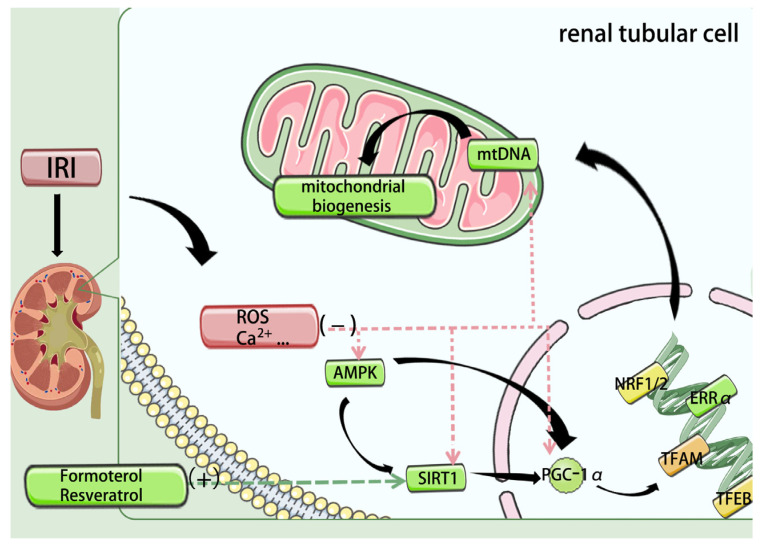
Upon activation by the AMPK/SIRT1 signaling pathway, PGC-1α functions as a transcriptional coactivator to upregulate the expression of key transcription factors, including NRF1, NRF2, ERRα, TFEB and TFAM, thereby collectively promoting mitochondrial biogenesis. PGC-1α: peroxisome proliferator–activated receptor-γ coactivator-1α; NRF1/2: nuclear respiratory factor-1/2; ERRα: estrogen-related receptor-α; TFAM: mitochondrial transcription factor A; TFEB: transcription factor EB. Additional mechanistic note: AMPK→SIRT1 deacetylation of PGC-1α promotes NRF1/2–TFAM-driven mtDNA replication and OXPHOS gene expression, while inflammatory signaling can suppress this biogenesis checkpoint.

Mitochondrial biogenesis is the process by which cells generate new mitochondria to adjust organelle number and quality, thereby augmenting cellular bioenergetic capacity and fitness [53]. This multilayered, highly coordinated program is governed by the transcriptional coactivator peroxisome proliferator–activated receptor-γ coactivator-1α (PGC-1α) and its downstream transcriptional network [54]. In ischemia–reperfusion-induced AKI, sustained downregulation of PGC-1α has been documented [55]. As a coactivator, PGC-1α drives the expression of a panel of nuclear genes—including nuclear respiratory factor-1 (NRF1), nuclear respiratory factor-2 (NRF2), estrogen-related receptor-α (ERRα), TFAM, and transcription factor EB (TFEB)—to coordinate mitochondrial biogenesis [56,57,58]. Upstream, AMPK/SIRT1 signaling tightly couples cellular energy status to PGC-1α activation and thereby to glucose metabolism and fatty-acid oxidation [59]; notably, AMPK directly phosphorylates PGC-1α at Thr177 and Ser538, enhancing its activity [60]. Conversely, p53 activation in IRI has been linked to repression of PGC-1α expression [55] (Figure 2).

Among PGC-1α targets, Nrf2 and ERRα transcriptionally regulate numerous respiratory-chain genes [56,61]. In addition, Nrf2—a distinct antioxidant master regulator—modulates inflammatory and oxidative responses in AKI; genetic or pharmacologic inhibition of Nrf2 exacerbates inflammation and oxidative stress in IRI-AKI models [62,63]. TFAM is indispensable for mtDNA transcription and replication, acting downstream of NRF1/2 [64,65]; in ischemic AKI, TFAM inhibition reduces mtDNA expression and aggravates renal injury [17]. TFEB can directly bind promoters to regulate PGC-1α and NRF1/2 expression, thereby influencing the biogenesis program [66]. Conceptually, therapeutic enhancement of mitochondrial biogenesis in renal IRI aims to restore the PGC-1α-centered transcriptional axis.

A growing body of evidence supports this strategy. In oxidant-challenged renal proximal tubular cells (RPTCs), PGC-1α overexpression accelerates recovery of mitochondrial and cellular function [67]. Accordingly, interventions that amplify AMPK/SIRT1/PGC-1α signaling have garnered attention. Our group showed that inhibition of microRNA-132-3p preserves its target SIRT1 and mitigates ischemic AKI [68]. BMAL1 has also been identified as a protective regulator of the SIRT1/PGC-1α axis in renal IRI [69]. Mechanistically, tubular CD44 represses PGC-1α transcription via NF-κB p65; thus, targeting CD44 emerges as a promising therapeutic approach [70]. Pharmacologic inducers of biogenesis—including the 5-HT1F agonist lasmiditan and the β2-adrenergic agonist formoterol—have shown potential to promote biogenesis, attenuate IRI, and limit fibrosis in preclinical models [71,72]. The SIRT1 agonist resveratrol has attracted increasing interest in IRI; a systematic review and meta-analysis of 19 studies reported significant reductions in serum creatinine (SCr) and blood urea nitrogen (BUN) in renal IRI models [69,73,74]. Finally, activating downstream transcription factors, especially Nrf2 with its anti-inflammatory and antioxidant actions, is gaining traction as a complementary strategy to reinforce the biogenesis program [75].

### 5.2. Mitochondrial Dynamics

Maintenance of mitochondrial function depends on dynamic equilibrium. Mitochondrial dynamics—the continual remodeling of the network by fission and fusion—sustains this equilibrium and is therefore essential for bioenergetic homeostasis, stress responses, and apoptosis control [76].

**Figure 3:**
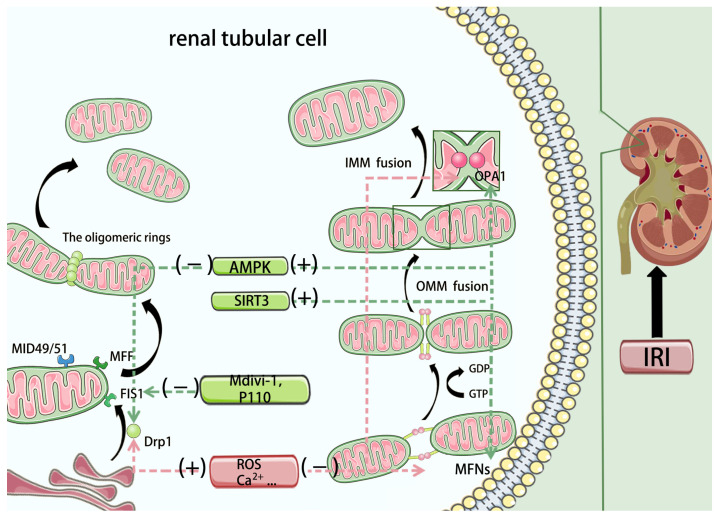
Mitochondrial dynamics represent a precisely regulated cyclic process. Fission is primarily mediated by Drp1, which is recruited to the mitochondrial outer membrane by specific receptor proteins (such as MFF, FIS1, MID49/51) and subsequently assembles into oligomeric rings that constrict and sever the mitochondrion. Fusion, in contrast, occurs as a sequential two-step event: MFN1/2 drive outer membrane fusion through nucleotide-dependent conformational changes, while OPA1, existing in a specific equilibrium of long and short isoforms, mediates inner membrane fusion and remodels cristae architecture. Drp1: dynamin-related protein-1; FIS1: fission protein 1; MFF: mitochondrial fission factor; MID49/51: mitochondrial dynamics proteins49/51; MFN1/2: mitofusin-1/2; OPA1: optic atrophy-1. Additional mechanistic note: Stress-activated Drp1 recruitment and OPA1 processing shift the balance toward fragmentation, which can facilitate mPTP opening, cytochrome c release, and downstream regulated cell death.

Fission (Figure 3). Mitochondrial fission is governed primarily by dynamin-related protein-1 (Drp1), whereas fusion is controlled by mitofusin-1/2 (MFN1/2) and optic atrophy-1 (OPA1) [50]. Recruitment of Drp1 to defined fission sites on the outer mitochondrial membrane (OMM) is a key initiating event [77]. Because Drp1 lacks a canonical phospholipid-binding domain, OMM “adapters”—FIS1, MFF, and the mitochondrial dynamics proteins MiD49/MiD51—engage Drp1 via its G domain and stalk to position and anchor it on the OMM [78,79]. Drp1 activation at this stage is finely tuned by post-translational modifications (PTMs)—including phosphorylation/dephosphorylation, ubiquitination, and SUMOylation. Once recruited, Drp1 self-assembles into oligomeric rings/spirals; as a large GTPase of the dynamin family, it hydrolyzes GTP to constrict and sever the membrane [80]. Final scission has been attributed, at least in part, to the recruitment of dynamin-2 (DNM2) [81]. Current evidence further suggests that inner mitochondrial membrane (IMM) fission can proceed independently, mediated by short OPA1 (S-OPA1) and the IMM protein MTP18 [82].

Fusion (Figure 3). Mitochondrial fusion proceeds in two sequential steps—OMM fusion, then IMM fusion [83]. The highly homologous GTPases MFN1 and MFN2 drive OMM fusion: GTP binding converts MFNs from a conserved folded conformation into an extended, “upright” state; MFNs on opposing mitochondria then form trans homo- or hetero-oligomers through G-domain contacts, and GTP hydrolysis drives outer-membrane merger [84,85]. Despite homology, MFN1 exhibits higher GTPase activity, whereas MFN2 oligomers are more stable [86,87]. IMM fusion requires OPA1. The long isoform (L-OPA1) is anchored in the IMM via an N-terminal transmembrane segment; regulated cleavage by YME1L (i-AAA protease) and OMA1 (metallopeptidase) generates S-OPA1 [82,88]. An appropriate L-OPA1:S-OPA1 ratio and subsequent conformational transitions are necessary to execute IMM fusion [89]. Beyond fusion, L-OPA1 is pivotal for cristae remodeling and ultrastructural integrity [90].

Pathological remodeling in IRI. During ischemia–reperfusion, accumulating ROS and Ca^2+^ promote Drp1 dephosphorylation and translocation to mitochondria, driving excessive fission and fragmentation—a key amplifier of renal injury [91,92,93]. Genetic ablation of Drp1 or pharmacologic inhibition with Mdivi-1 reduces fragmentation and injury in preclinical models, though each approach has limitations [94,95]. By contrast, our group reported that P110, a selective peptide that disrupts the Drp1–FIS1 interaction (functionally restraining pathological fission), attenuates mitochondrial damage in renal IRI and shows translational promise [96]. Among Drp1 PTMs, Ser616 phosphorylation correlates with pro-fission recruitment, whereas Ser637 phosphorylation suppresses Drp1 GTPase activity [97]. We also showed that microRNA-199a-5p targets AKAP1, promoting Drp1-Ser637 dephosphorylation, thereby exacerbating mitochondrial fragmentation; conversely, inhibiting miR-199a-5p confers renoprotection [98].

On the fusion axis, MFN2 and OPA1 expression is frequently downregulated in IRI, contributing to network disconnection and injury propagation [99,100]. The deacetylase SIRT3 has emerged as a regulator that stabilizes fusion: in ischemia–reperfusion models, SIRT3 overexpression mitigates damage by inhibiting MFN2 ubiquitination and/or modulating OPA1 isoform balance, delaying the shift toward fission [101,102]. Additional candidate targets for rebalancing dynamics include tissue inhibitors of metalloproteinases (TIMPs), the purinergic receptor P2RX1, and TRIM35 [103,104,105]. Upstream, AMPK not only promotes biogenesis but also fine-tunes fission–fusion. For example, empagliflozin activates AMPK and upregulates OPA1, suppressing mitochondrial shortening/fragmentation in HK-2 cells [106]; melatonin preconditioning similarly engages an AMPK/Drp1 pathway to alleviate oxidative injury [107].

Beyond direct manipulation of fission/fusion proteins, mitochondrial dynamics is regulated across transcriptional, post-transcriptional, and post-translational layers. For example, non-coding RNAs (e.g., miR-199a-5p) can reprogram Drp1-driven fission, while phosphorylation and other PTMs of Drp1 integrate stress cues to dictate mitochondrial morphology [97,98]. Therefore, therapeutic approaches may target upstream signaling or epigenetic regulators in addition to direct fission inhibitors such as Mdivi-1 and P110 [94,95,96]. Importantly, most modulators of mitochondrial dynamics remain preclinical in renal IRI-AKI, and future work must address specificity, off-target effects, and the optimal treatment window for dynamic remodeling.

In sum, therapeutically rebalancing mitochondrial fission and fusion is a compelling strategy for renal IRI—one that complements biogenesis and mitophagy arms of mitochondrial quality control and has yielded encouraging preclinical signals.

### 5.3. Mitophagy

As a form of selective autophagy, mitophagy eliminates damaged or dysfunctional mitochondria to maintain network integrity. Under basal conditions, continual cycles of fusion and fission renew the mitochondrial population, while mitophagy removes organelles that are excessively fragmented, depolarized, or overproduce ROS, thereby sustaining mitochondrial health [34].

**Figure 4:**
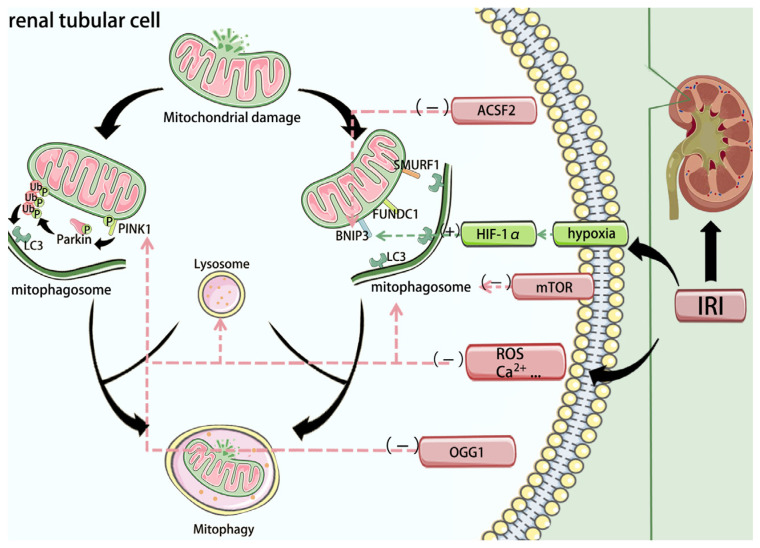
Mitophagy is primarily mediated through two distinct pathways. The first is the PINK1/Parkin-dependent pathway, where mitochondrial damage stabilizes PINK1 on the outer mitochondrial membrane, leading to recruitment and activation of Parkin. Parkin then ubiquitinates mitochondrial proteins, promoting autophagy receptor recruitment and mitophagy initiation. The second involves receptor-mediated pathways, where outer membrane proteins such as BNIP3, FUNDC1, and SMURF1—upregulated under hypoxia—directly interact with LC3 on the phagophore membrane, targeting damaged mitochondria for autophagic degradation. PINK1: PTEN-induced kinase 1; BNIP3: BCL2 interacting protein 3; FUNDC1: FUN14 domain-containing 1; SMURF1: Smad specific E3 ubiquitin protein ligase 1. Additional mechanistic note: Completion of mitophagy requires efficient autophagosome–lysosome fusion and lysosomal degradation; impaired flux can lead to persistence of damaged mitochondria and sustained mtDAMP-driven inflammation.

Mitophagy proceeds through two principal routes: a ubiquitin-dependent pathway driven by PINK1/Parkin, and receptor-mediated pathways on the outer mitochondrial membrane (OMM). In healthy mitochondria, PTEN-induced kinase 1 (PINK1) is constitutively imported and cleaved by the IMM protease PARL (presenilin-associated rhomboid-like protein), followed by cytosolic degradation; thus PINK1 remains low at steady state [108]. When the mitochondrial membrane potential collapses, PINK1 accumulates on the OMM, recruits and phosphorylates cytosolic Parkin (E3 ligase), and activates it on the mitochondrial surface. Parkin-dependent ubiquitination of OMM proteins then recruits LC3-binding autophagy receptors, initiating mitophagosome formation [109]. In renal IRI, PINK1–Parkin-mediated mitophagy is robustly induced; conversely, genetic or pharmacologic impairment of PINK1/Parkin aggravates mitochondrial injury in HK-2 cells, indicating a protective role for this pathway in IRI [110] (Figure 4).

In receptor-mediated mitophagy, OMM proteins such as BNIP3, FUNDC1, and SMURF1 serve as molecular tethers to the autophagy machinery [111]. Upon specific cues (e.g., hypoxia), these receptors are upregulated and directly bind LC3 via LIR motifs to target compromised mitochondria for clearance [112]. For example, HIF-1α transcriptionally induces BNIP3 under hypoxia, embedding it in the OMM to drive mitophagy. In mice, HIF-1α depletion leads to BNIP3 downregulation weakens post-ischemic mitophagy, leading to accumulation of damaged mitochondria and worse renal injury; BNIP3 overexpression enhances mitophagy, limits mtROS, and improves ischemic AKI outcomes [113,114].

Given that IRI induces widespread mitochondrial depolarization and oxidative stress, timely initiation of mitophagy is crucial to prevent injury escalation. Therapeutically, preserving or fine-tuning mitophagy is therefore attractive. In our work, 8-oxoguanine DNA glycosylase (OGG1) acted as a negative regulator of PINK1/Parkin-dependent mitophagy, exacerbating ischemic damage; OGG1 knockout or pharmacologic inhibition mitigated apoptosis in vitro and protected kidneys in IRI models [115]. Upstream of BNIP3, ACSF2 (acyl-CoA synthetase family member-2) has been identified as a regulatory node: ACSF2 knockdown enhanced autophagy and attenuated IRI-induced renal injury [116]. In proximal tubules, FUNDC1 deletion suppresses mitophagy and over-activates Drp1-dependent fission, amplifying inflammation and cell death [117]. Consistent with a hypoxia-adaptive program, roxadustat—a HIF prolyl-hydroxylase inhibitor that stabilizes HIF-1α—can enhance mitophagy via the HIF-1α/FUNDC1 axis and protect against IRI [118].

Importantly, mitophagy in AKI appears context- and dose-dependent: moderate activation is beneficial for removing damaged mitochondria, whereas excessive mitophagy may deplete mitochondrial mass and precipitate an energy crisis [119]. Mechanistically, loss of PANX1 was shown to relieve mTORC1-mediated suppression of autophagy, thereby enhancing mitophagy and improving removal of injured mitochondria [120]. By contrast, in our study caloric restriction suppressed mTORC1 and over-activated mitophagy, which—despite efficient clearance—worsened renal outcomes in IRI [121]. This is considered to be related to the role of mitophagy as a protective mechanism during ischemia: in the early phase, it clears damaged mitochondria and prevents the accumulation of harmful metabolites; however, during reperfusion, if autophagic activity is excessively activated, it may lead to the accumulation of undegraded materials within autolysosomes, thereby potentially causing cellular injury. Clinically, this implies that mitophagy modulation requires a definable therapeutic window. A practical path forward is to couple mitophagy-targeted therapy with synchronous monitoring of mitochondrial injury. This requires integrated analysis of indicators reflecting “clearance efficiency” (e.g., autophagy markers like LC3-II and mTOR signaling molecules [122]) and those reflecting “mitochondrial health/quantity” (e.g., urinary mitochondrial DNA as a non-invasive marker of mitochondrial disruption [123,124]). Where feasible, this should be supplemented with functional imaging of regional mitochondrial activity (e.g., complex-I PET [30]) (Additional clinical detection indicators will be introduced below). This combined approach ensures that the enhancement of autophagic activity does not lead to the depletion of functional mitochondrial mass, thereby enabling precise control of the therapeutic window. Such biomarker-guided titration could help identify patients who would benefit from mitophagy enhancement (to clear damaged organelles) versus those in whom further activation could exacerbate energy failure and delay recovery. In addition, prolonged mitophagy stimulation may need to be paired with mitochondrial biogenesis support to replenish organelle mass during the recovery phase [125]. Therefore, for therapies targeting mitophagy, mastering the appropriate degree and timing of autophagic intervention is critical.

## 6. Release of DAMPs and Associated Immune Mechanisms

Beyond their bioenergetic role, mitochondria are pivotal immunoregulatory organelles. During renal ischemia–reperfusion injury (IRI), damaged mitochondria release multiple mitochondria-derived damage-associated molecular patterns (mtDAMPs)—including mtDNA, mtRNA, and cardiolipin—which are sensed by cellular surveillance systems to activate innate immune pathways, amplifying tissue injury and propagating systemic inflammation [126].

mtDNA is the prototypical mtDAMP. Owing to features that resemble microbial DNA, mtDNA engages pattern-recognition receptors (PRRs) and triggers downstream inflammatory programs, notably TLR9, cGAS–STING, and inflammasome signaling, thereby initiating innate immune responses that drive both local renal and systemic injury [127,128,129]. TLR9, an endosomal receptor for unmethylated CpG motifs [130], readily recognizes mtDNA because mitochondria retain CpG-rich, hypomethylated sequences from their bacterial ancestry, rendering mtDNA structurally similar to PAMPs [131,132]. When mtDNA gains access to endosomes in tubular or immune cells, TLR9 signals via MyD88, activating MAPK and NF-κB pathways and inducing cytokines such as TNF-α and IL-6 [133]. In ischemic AKI models, tubule-specific TLR9 deletion mitigates IRI-AKI, highlighting TLR9 as a potential anti-inflammatory target [134].

Cytosolic self-DNA—including leaked mtDNA—activates cGAS, which synthesizes cGAMP to bind and activate STING at the endoplasmic reticulum; activated STING recruits TBK1 to phosphorylate IRF3, inducing type-I interferons and pro-inflammatory mediators [135,136]. Notably, the STING inhibitor H-151 blocks this pathway and confers robust protection in renal IRI [137]. In parallel, leaked/oxidized mtDNA can activate intracellular inflammasome complexes. The NLRP3 inflammasome—comprising NLRP3, ASC, and caspase-1—is the best characterized: oxidized mtDNA binds NLRP3, promotes its conformational activation and assembly, and drives caspase-1–dependent maturation of IL-1β and IL-18 [138,139]. These axes collectively position mtDNA sensing as a key amplifier of post-ischemic inflammation and an attractive therapeutic entry point.

Therapeutic strategies can also act upstream to curtail mtDNA release and enhance its clearance. Inhibiting mPTP opening with the cyclophilin-D (CypD) inhibitor cyclosporin A (CsA) stabilizes the mitochondrial membrane potential (ΔΨm) and reduces tubular apoptosis, underscoring the value of targeting CypD in IRI [140]. Enzymatic degradation of extracellular DNA with DNase I diminishes renal injury in rat IRI, implicating circulating cell-free DNA (including mtDNA) as a tractable driver of damage [141]. Augmenting mitophagy accelerates removal of dysfunctional mitochondria and limits mtDNA leakage at the source [142]. Clinically, mtDNA also holds biomarker potential: in mice, plasma mtDNA rises after renal ischemia and correlates with injury severity; in kidney-transplant recipients, ischemia time correlates with postoperative urinary extracellular mtDNA, suggesting urinary mtDNA as a noninvasive indicator of AKI risk and severity [143].

Recent spatial transcriptomic and single-cell studies reveal marked spatial heterogeneity in mitochondrial-immune interactions during kidney injury. Different immune cell subsets exhibit distinct mitochondrial metabolic states within specific renal anatomical regions, closely correlating with injury severity. The kidney’s cortico-medullary oxygen gradient, nephron-segment specialization, and spatially restricted mitochondrial architecture generate ‘danger niches’ where mtDAMP release and immune activation are concentrated. Particularly in the outer medulla, regions enriched with mtROS show dense immune cell signals and enrichment of cell death pathways, suggesting the existence of “metabolic-immune hot spots” [27]. Recent work has begun mapping injury-specific microenvironments and cellular interactions in kidney regeneration and disease, revealing spatially dependent signaling among persistently injured tubules, stromal cells, and immune populations [144,145,146]. Liu et al. identified, via spatial neighborhood analysis, a novel neutrophil-centric niche characterized by interactions between neutrophils and thick ascending limbs (TAL) [147]. Another study showed macrophages and dendritic cells localized within distinct microenvironments, with their spatial distribution dynamically shifting after bilateral ischemia–reperfusion injury [148]. Meanwhile, scRNA-seq have further unveiled temporal heterogeneity among immune cells. A study has found that macrophage infiltration increases markedly on the first day after AKI, followed by a second peak on day 14. During this process, a subset of M1 macrophages begins transitioning toward an M2-like state, suggesting that the restoration of metabolic-immune balance represents a critical checkpoint for renal repair [149,150]. These spatiotemporal patterns highlight renal IRI as a mitochondria-driven, dynamically amplified inflammatory process that operates across diverse cell types and over extended time scales. Complementary evidence from kidney transplantation illustrates the power of spatial immunology to resolve innate immune organization: transcriptional and spatial profiling of allografts has identified compartmentalized FcyRIII (FCGR3A)+ innate immune cells and linked their distribution to intragraft inflammation severity [151]. Spatial transcriptomics of human rejection biopsies further supports distinct monocyte/macrophage states with high FCGR3A expression and niche-specific programs, emphasizing that immune subsets occupy specific renal regions [152]. Applying these spatial frameworks to IRI-AKI—together with mitochondrial functional imaging and mitochondrial biomarkers—may clarify where and when mtDAMP signaling dominates and may inform targeted delivery and phase-specific combination therapy.

## 7. Mitochondria-Targeted Therapies for IRI-AKI

Translation of mitochondria-targeted interventions requires clinically feasible tools to (i) identify mitochondrial-driven AKI endotypes, (ii) determine the optimal therapeutic window, and (iii) monitor target engagement. Among candidate markers, urinary mitochondrial DNA (UmtDNA) has emerged as a non-invasive readout of mitochondrial injury; it correlates with renal dysfunction in AKI and reflects mitochondrial disruption [123,124]. Extracellular/urinary mtDNA has also been proposed as a marker of graft injury and delayed graft function, linking mtDAMP release to clinical outcomes [143]. Beyond mtDNA, panels that combine mitochondrial biomarkers with conventional tubular injury markers (e.g., NGAL, KIM-1) may improve patient stratification. The UmtDNA field encompasses measurements of mtND1/mtCOXIII copy number, mitochondrial transcripts, and extracellular vesicle cargo, although standardization of sampling timepoints, normalization methods, and cut-offs remains a major challenge [124]. Imaging modalities provide spatial context. Complex-I PET imaging (18F-BCPP-BF) detects early and region-specific loss of mitochondrial function after renal reperfusion—often preceding changes in total mitochondrial protein abundance—suggesting a role for functional imaging in early risk stratification and in evaluating mitochondrial drug responses [30]. Integrating longitudinal biomarkers with imaging and clinical covariates could enable biomarker-guided trials of mitochondria-targeted therapy in IRI-AKI.

A multi-axis therapeutic framework has emerged for mitochondrial targeting in renal ischemia–reperfusion injury (IRI). Key strategies include: (i) deploying antioxidants (e.g., MitoQ, SS-31) to quench excess ROS; (ii) activating the AMPK/SIRT1/PGC-1α axis to stimulate mitochondrial biogenesis; (iii) rebalancing dynamics by inhibiting Drp1-dependent fission or enhancing MFN2/OPA1-mediated fusion; (iv) fine-tuning mitophagic flux (e.g., PINK1/Parkin and related pathways) to preserve network quality; and (v) interrupting mtDAMP sensing, for example with TLR9 or cGAS–STING pathway inhibitors, to blunt downstream inflammatory cascades. In combination, these layers enable coordinated control of redox injury, organelle turnover, and sterile inflammation, and together have produced robust renoprotective signals in preclinical models. Current application of mitochondria-targeted drugs in IRI-AKI continues to face a series of critical challenges. A primary bottleneck lies in delivery efficiency, as achieving sufficient mitochondrial enrichment within vulnerable nephron segments without incurring off-target toxicity remains difficult. Furthermore, most compounds lack precise specificity, frequently perturbing multiple pathways—such as redox cycling or membrane potential regulation—which can lead to unintended effects. The therapeutic window is notably narrow, with efficacy largely confined to a brief ischemia/reperfusion period, while administration outside this window may inhibit endogenous adaptive repair responses. Significant heterogeneity in mitochondrial phenotypes and local immune microenvironments across different renal cell types also complicates intervention, likely requiring tailored or stratified therapeutic strategies. Moreover, prolonged modulation of fundamental processes like mitochondrial quality control or innate immune signaling carries the risk of impairing host defense mechanisms or disrupting mitochondrial homeostasis. Overcoming these hurdles will probably necessitate biomarker-guided dynamic dosing, rationally designed combination regimens, and the development of standardized pipelines for clinical translation.

An additional, rapidly advancing modality is mitochondrial transfer, whereby functional mitochondria move between cells or to specific subcellular locales. By delivering healthy mitochondria to injured cells, this approach can compensate for bioenergetic failure and promote tissue repair, and is increasingly viewed as a novel avenue for AKI therapy [153,154]. Mesenchymal stem cells (MSCs) have been shown to transfer mitochondria to damaged tubular epithelial cells, augmenting respiratory-chain activity and ATP generation [155,156]. In particular, Perico et al. demonstrated that human umbilical-cord MSCs (UC-MSCs) implanted into cisplatin-injured kidneys increased tubular mitochondrial content and restored function [156].

Beyond endogenous intercellular transfer, artificial mitochondrial transplantation provides direct organelle replacement by isolating functional mitochondria from donor cells and delivering them—via intravenous/arterial perfusion or local injection—to ischemic tissue [157]. In a porcine IRI-AKI model, Doulamis et al. infused autologous mitochondria through the renal artery, achieving substantial in vivo mitochondrial transfer; treated animals exhibited higher glomerular filtration rates, lower serum creatinine and BUN, and reduced histologic injury versus controls [158]. Consistently, an ex vivo porcine kidney study reported milder pathologic injury after mitochondrial transplantation compared with untreated organs [159]. Although mitochondrial transplantation therapy demonstrates revolutionary potential, its safety and feasibility in large animals and even humans require thorough validation [160]. Ensuring high mitochondrial integrity and functional activity is considered a prerequisite for therapeutic success [161]. However, current efficacy relies on freshly isolated, respiration-competent mitochondria, and their activity declines rapidly post-isolation. While existing cryopreservation techniques can maintain structural integrity, they compromise function [162,163]. Therefore, future efforts must focus on establishing Good Manufacturing Practice (GMP) standards, developing efficient and non-destructive isolation and storage technologies, and deeply investigating the interaction mechanisms between transplanted mitochondria and recipient cells. These steps are crucial to advance this promising strategy for overcoming intractable acute kidney injury toward clinical application.

## 8. Conclusions

Mitochondria occupy a central position in the pathogenesis and progression of renal IRI. This review synthesizes the principal mechanisms of mitochondrial dysfunction in IRI-AKI and outlines therapeutic strategies that target these nodes—antioxidant defenses, biogenesis, dynamics, mitophagy, and mtDAMP signaling—which collectively show strong promise in preclinical studies (Table 2).

Important limitations remain. Most mechanistic insights derive from cell and animal models, and clinical translatability must be established. The specificity, safety, and delivery of mitochondria-targeted agents require optimization, and the integrative network control among quality-control modules (biogenesis–dynamics–mitophagy–inflammation) is not yet fully resolved. Future work should map these regulatory networks in greater depth within IRI, define dose–time windows for pathway modulation, and advance well-designed clinical trials to translate mitochondria-targeted interventions into practice. Moreover, to enable precision mitochondria-targeted therapy, several key knowledge gaps must be addressed, including: resolving cell-type and nephron-segment specific mitochondrial phenotypes and their temporal trajectories using integrated single-cell/spatial omics and functional imaging; establishing clinically feasible mitochondrial biomarkers (e.g., urinary mtDNA-based panels) for patient stratification and target engagement monitoring; delineating bidirectional mitochondrial–immune crosstalk and spatial ‘danger niches’ that shape sterile inflammation; and optimizing delivery platforms and combination regimens that simultaneously support redox balance and mitochondrial quality control while avoiding immune suppression.

## Figures and Tables

**Table 1:** Representative compounds/approaches targeting mitochondrial antioxidant defense and redox injury in renal IRI-AKI.

Agent/Approach	Primary Target/Mechanism	Evidence in Renal IRI-AKI Models	Notes/Translational Considerations
NOX inhibition	Suppresses NOX-derived ROS and downstream oxidative stress	Selective NOX inhibition reduced ROS overproduction and attenuated renal injury in IRI models [39,40]	Preclinical; may complement mitochondria-targeted antioxidants.
Remote ischemic preconditioning (rIPC)	Dampens NOX4–ROS signaling; limits ferroptosis	Reported to mitigate mitochondrial dysfunction and ferroptosis during AKI [41]	Non-pharmacologic; timing and patient selection are critical.
MitoQ/SkQ/CoQ10/curcumin	Mitochondria-directed or mitochondrial-supportive redox scavengers; preserve ETC and membranes	Shown to reduce mtROS and improve renal outcomes in experimental IRI/AKI [43,44,45]	Dose and delivery determine mitochondrial accumulation and efficacy.
SS-31 (elamipretide)	Binds cardiolipin to stabilize the inner mitochondrial membrane; lowers mtROS	Markedly lowers mtROS and stabilizes mitochondrial architecture in ischemic AKI models [46,47]	Translational candidate; requires optimization of dosing window.
SOD2 mRNA-LNP	Restores mitochondrial antioxidant enzyme activity (SOD2)	LNP delivery of chemically modified SOD2 mRNA conferred renoprotection in murine renal IRI [48]	Nucleic-acid therapy; delivery and immunogenicity need evaluation.
Thylakoid–liposome + L-ascorbic acid (ultrasound-assisted renal targeting)	Enhances antioxidant capacity and tubular bioenergetics	Improved renal function and restored tubular bioenergetics in experimental AKI [49]	Platform approach; requires device-assisted targeting and scalability studies.

**Table 2:** Abbreviations: AKI, acute kidney injury; ETC, electron transport chain; IMM, inner mitochondrial membrane; IRI, ischemia–reperfusion injury; LNP, lipid nanoparticle; mPTP, mitochondrial permeability transition pore; mtDAMP, mitochondria-derived damage-associated molecular pattern; mtROS, mitochondrial reactive oxygen species; NOX, NADPH oxidase.

Therapeutic Axis	Representative Agent(s)/Intervention(s)	Primary Target(s)/Pathway(s)	Key Proposed Mechanism(s) in IRI-AKI	Evidence Level (as Reported)
mtROS scavenging (mitochondria-directed antioxidants)	MitoQ; SkQ; CoQ10; curcumin	Mitochondrial redox buffering/CoQ analogs	Quench mtROS, limit lipid peroxidation and ETC damage, preserve membrane potential	Preclinical (cell/animal IRI-AKI models) [43,44,45]
Inner mitochondrial membrane stabilization	SS-31 (elamipretide)	Cardiolipin/IMM architecture	Binds cardiolipin to stabilize cristae/IMM structure, lowers mtROS and improves ATP recovery	Preclinical (ischemic AKI models) [46,47]
Augment endogenous antioxidant enzymes (RNA therapy)	LNP-delivered chemically modified SOD2 mRNA	SOD2 (mitochondrial MnSOD)	Boost superoxide clearance to blunt oxidative injury and preserve mitochondrial function	Preclinical (murine renal IRI) [48]
Targeted antioxidant delivery platform	Ultrasound-responsive thylakoid-integrating liposomes loaded with L-ascorbic acid	Tubular bioenergetics/antioxidant capacity	Spatially triggered delivery; restores mitochondrial repair programs and improves renal function	Preclinical (experimental AKI) [49]
Reduce ROS production (NOX inhibition)	NOX1 inhibitor; apocynin (NOX-associated)	NOX family-derived ROS	Suppress NADPH oxidase-driven ROS overproduction and downstream ERK/oxidative injury	Preclinical (cell/animal) [39,40]
Remote ischemic preconditioning (rIPC)	rIPC	NOX4-ROS signaling; ferroptosis axis	Dampens NOX4-driven ROS, mitigates mitochondrial dysfunction and ferroptosis in tubular cells	Preclinical [41]
Mitochondrial biogenesis induction	Lasmiditan	AMPK/SIRT1/PGC-1α axis	Promotes mitochondrial biogenesis to accelerate recovery of tubular mitochondrial function after AKI	Preclinical [71]
Mitochondrial biogenesis induction	Formoterol	β2-adrenergic receptor signaling (proximal tubule)	Enhances mitochondrial recovery and renal function after IRI	Preclinical [72]
Sirtuin activation/biogenesis support	Resveratrol	SIRT1/PGC-1α; antioxidant/anti-inflammatory networks	Enhances mitochondrial homeostasis and reduces functional injury markers in renal IRI models	Preclinical (meta-analysis of animal studies) [74]
Rebalance fission-fusion: inhibit excessive fission	Mdivi-1	Drp1-mediated fission	Limits pathological mitochondrial fragmentation to reduce tubular injury (noting limitations)	Preclinical [94,95]
Rebalance fission-fusion: block pathological fission interface	P110 peptide	Drp1-FIS1 interaction	Selectively suppresses stress-induced fission while sparing basal dynamics	Preclinical/translational potential [96]
Rebalance fission-fusion: promote fusion	Empagliflozin	AMPK-OPA1 pathway	Activates AMPK and upregulates OPA1 to suppress shortening/fragmentation	Preclinical (HK-2 cells; IRI context) [106]
Preconditioning to modulate dynamics and autophagy	Melatonin preconditioning	AMPK/Drp1; autophagy pathways	Reduces oxidative injury and restores dynamics-autophagy balance	Preclinical [107]
Fine-tune mitophagy (receptor-mediated)	Roxadustat (HIF prolyl hydroxylase inhibitor)	HIF-1α/FUNDC1 mitophagy axis	Enhances mitophagy to clear damaged mitochondria and limit ROS/inflammation	Preclinical [118]
Fine-tune mitophagy (PINK1/Parkin)	OGG1 inhibition/knockout (proof-of-concept)	PINK1/Parkin-dependent mitophagy	Relieves repression of mitophagy; reduces apoptosis and renal injury	Preclinical [115]
Block mtDAMP sensing	H-151	cGAS-STING pathway	Inhibits mtDNA-triggered innate immune signaling and downstream inflammation	Preclinical [137]
Prevent mtDAMP release (upstream)	Cyclosporin A (CsA)	Cyclophilin D/mPTP	Restrains pathological mPTP opening to stabilize ΔΨm and reduce apoptosis/mtDAMP leakage	Preclinical [140]
Enhance extracellular mtDNA clearance	DNase I	Cell-free DNA (incl. mtDNA)	Degrades extracellular DNA to dampen sterile inflammation and renal injury	Preclinical (rat IRI) [141]
Organelle replacement/transfer	MSC-mediated mitochondrial transfer; mitochondrial transplantation	Intercellular mitochondrial transfer/exogenous mitochondria delivery	Restores bioenergetics and promotes tubular repair; improves functional outcomes in large-animal models	Preclinical (rodent/pig) [155,156,158,159]

## Data Availability

No new data were created or analyzed in this study.
